# The effect of dendritic morphology on pattern recognition in the presence of active conductances

**DOI:** 10.1186/1471-2202-12-S1-P315

**Published:** 2011-07-18

**Authors:** Giseli de Sousa, Maex Reinoud, Adams Rod, Davey Neil, Steuber Volker

**Affiliations:** 1 Science and Technology Research Institute, University of Hertfordshire, Hatfield, Herts, AL10 9AB, UK

## 

In previous experiments [1], we showed that dendritic morphology affects the ability of passive neurons to recognise spatial patterns of synaptic inputs. In particular, the most symmetric morphologies outperformed the most asymmetric ones based on the measure of signal-to-noise ratio between stored and novel patterns. In the present study, we analyse how the dendritic morphology affects the pattern recognition performance in active models.

To evaluate pattern recognition performance, we ran a set of simulations using a large sample of neuronal morphologies each consisting of 128 terminal points and the same set of ion channel conductances, defined in previous models [2]. The model response was evaluated by calculating the signal-to-noise ratio over the number of spikes after presenting a pattern, differently from the experiments with passive models where the EPSP size was used [1,3]. For all experiments, we investigated whether the pattern recognition performance correlated with different morphometric parameters, including the asymmetry index [4], and the average and variance of path length. We also investigated suitable ranges for model parameters such as dendritic compartment length and synaptic strength, among other properties related to the pattern presentation. The results achieved in active models were then compared with the ones from passive models, using the same set of parameters.

**Figure 1:**
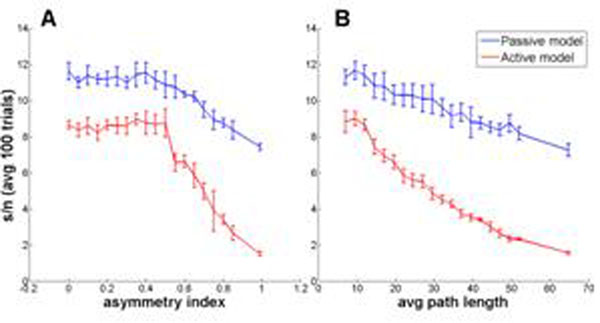
Pattern recognition performance in active and passive models. Results were obtained by generating a population of 150K random trees, and then averaging over 5 randomly selected ones in each bin, using two metrics: asymmetry index (**A**) and average path length (**B**).

The experiments confirmed that there are different pattern recognition abilities associated with a range of different morphologies from the most symmetric to the most asymmetric ones. The initial results suggested a strong anti-correlation between pattern recognition performance and neuronal asymmetry in the presence of active conductances (see Figure 1). The same correlation was also observed in passive models, however with a less accentuated performance difference when compared with active ones. The results also show that average path length is the best morphometric parameter tested to predict pattern recognition, where a more linear correlation was found when compared with other metrics (Figure 1B).

Currently we are investigating the optimization of active neuronal morphologies for pattern recognition, using an evolutionary algorithm, previously presented in [1]. In addition to the dendritic topology, different parameters were added to the genomic representation, such as dendritic compartment length and tapering, using the parameter ranges found in the previous experiments on these models. With these results, we want to confirm the more pronounced effect observed in active models (Figure 1), as they suggest the evolutionary algorithm may also be more successful in finding optimal morphologies for active as compared to passive dendrites.
